# Correction to “Association of adiposity indices with prehypertension among Chinese adults: A cross‐sectional study”

**DOI:** 10.1111/jch.14743

**Published:** 2023-11-29

Xiao M, Chen C, Wang J, et al. Association of adiposity indices with prehypertension among Chinese adults: A cross‐sectional study. *J Clin Hypertens* (Greenwich). 2023;25(5):470−479.

The authors have brought the following to our attention:

The corresponding author has been changed to: Yingqing Feng, Department of Cardiology, Guangdong Cardiovascular Institute, Guangdong Provincial People's Hospital, Guangdong Academy of Medical Sciences, Southern Medical University, Guangzhou 510080, China. Email: fengyingqing@gdph.org.cn

Mengyuan Xiao and Chaolei Chen contributed equally this work.

And finally, the third author affiliation has been updated as follows:

Department of Cardiology, Guangdong Cardiovascular Institute, Guangdong Provincial People's Hospital, Guangdong Academy of Medical Sciences, Southern Medical University, Guangzhou, China.

